# Supplementary material for: Impact of SDF-1 and AMD3100 on Hair Follicle Dynamics in a Chronic Stress Model
Source: Biomolecules. 2024 Sep 25;14(10):1206. doi: 10.3390/biom14101206 (PMC11505668; doi:10.3390/biom14101206)
Supplement: Supplementary file 1 [file biomolecules-14-01206-s001.zip › biomolecules-3076790-supplementary.pdf]

Table S1. siRNA sequence

Gene	Sequences
SDF-1	CTGAAGAACAACAACAGACAA
NC	CCAGATGGAACAGGACTAG

Table S2. RT-qPCR primer sequences

Gene	Sequences
SDF-1	Forward: 5'-TGCATCAGTGACGGTAAACCA-3'
	Reverse: 5'-CACAGTTTGGAGTGTTGAGGAT-3'
GAPDH	Forward: 5'-ACAGCAACAGGGTGGTGGAC-3'
	Reverse: 5'-TGAGGGTGCAGCGAACTT-3'

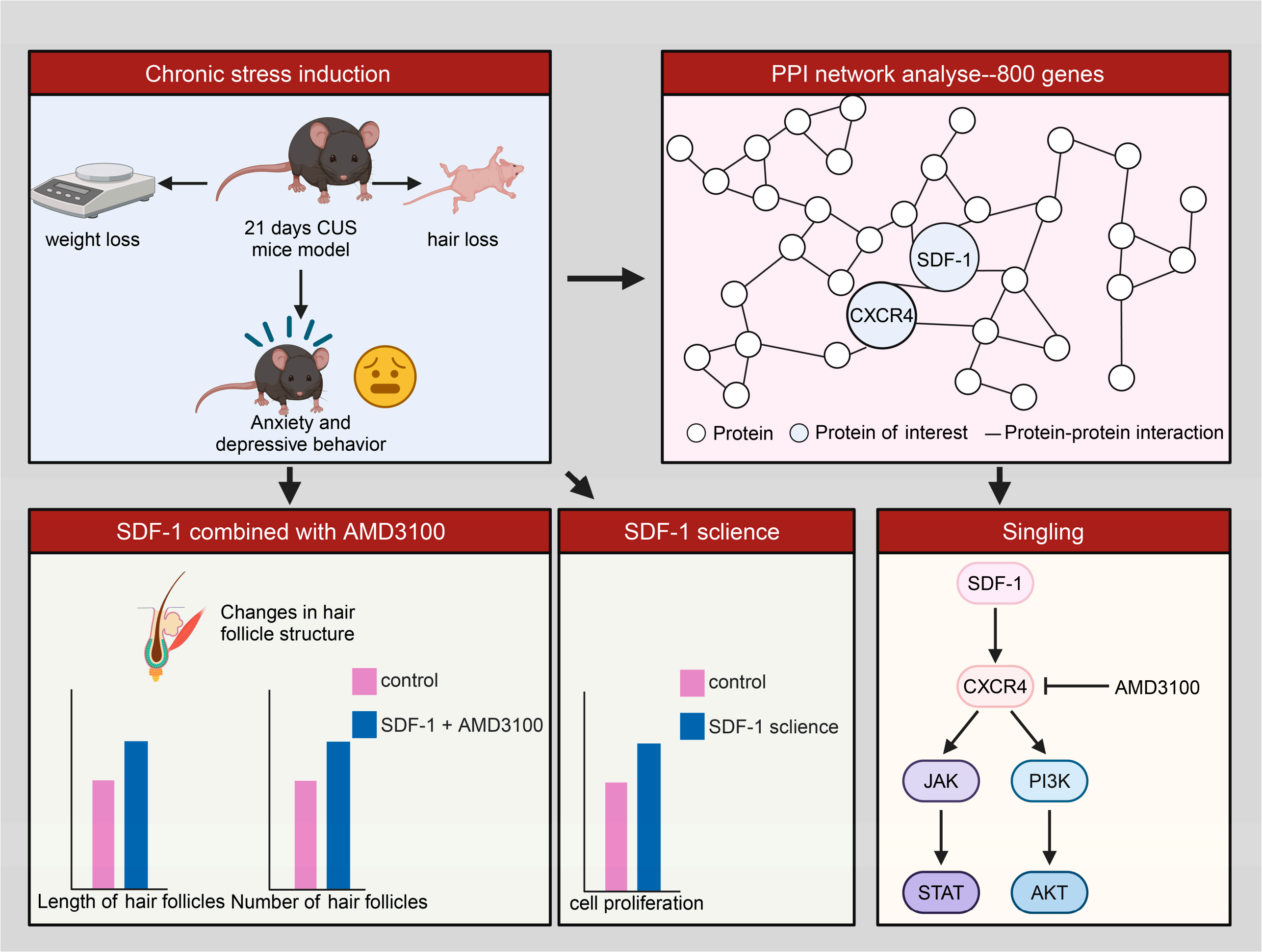

Figure S1. The experimental flow chart.
