# Supplementary figures and images for: Impact of SDF-1 and AMD3100 on Hair Follicle Dynamics in a Chronic Stress Model
Source: Biomolecules. 2024 Sep 25;14(10):1206. doi: 10.3390/biom14101206 (PMC11505668; doi:10.3390/biom14101206)

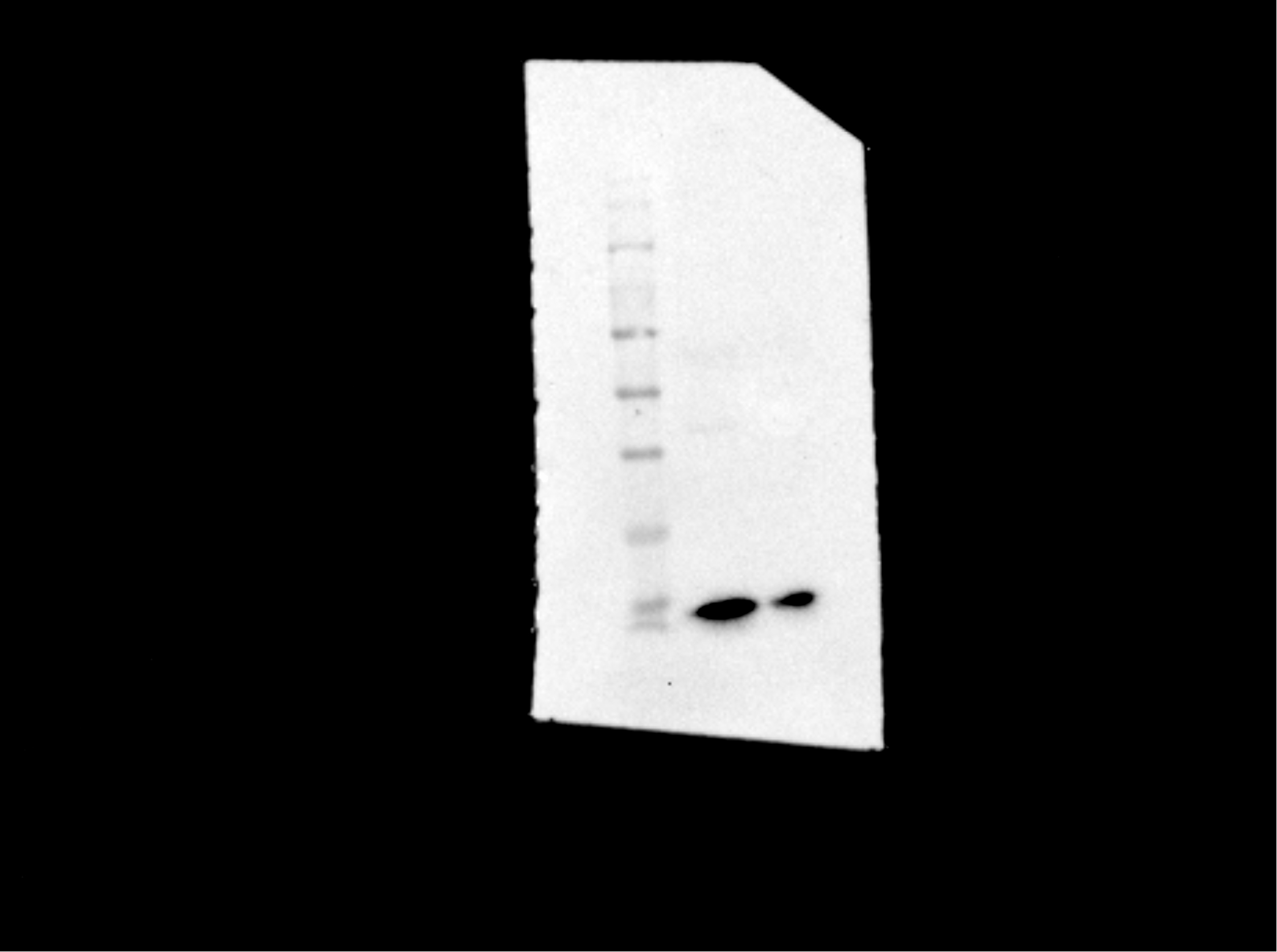

Supplement: Supplementary file 1 [file biomolecules-14-01206-s001.zip › Figure 6B-1-2.jpg]

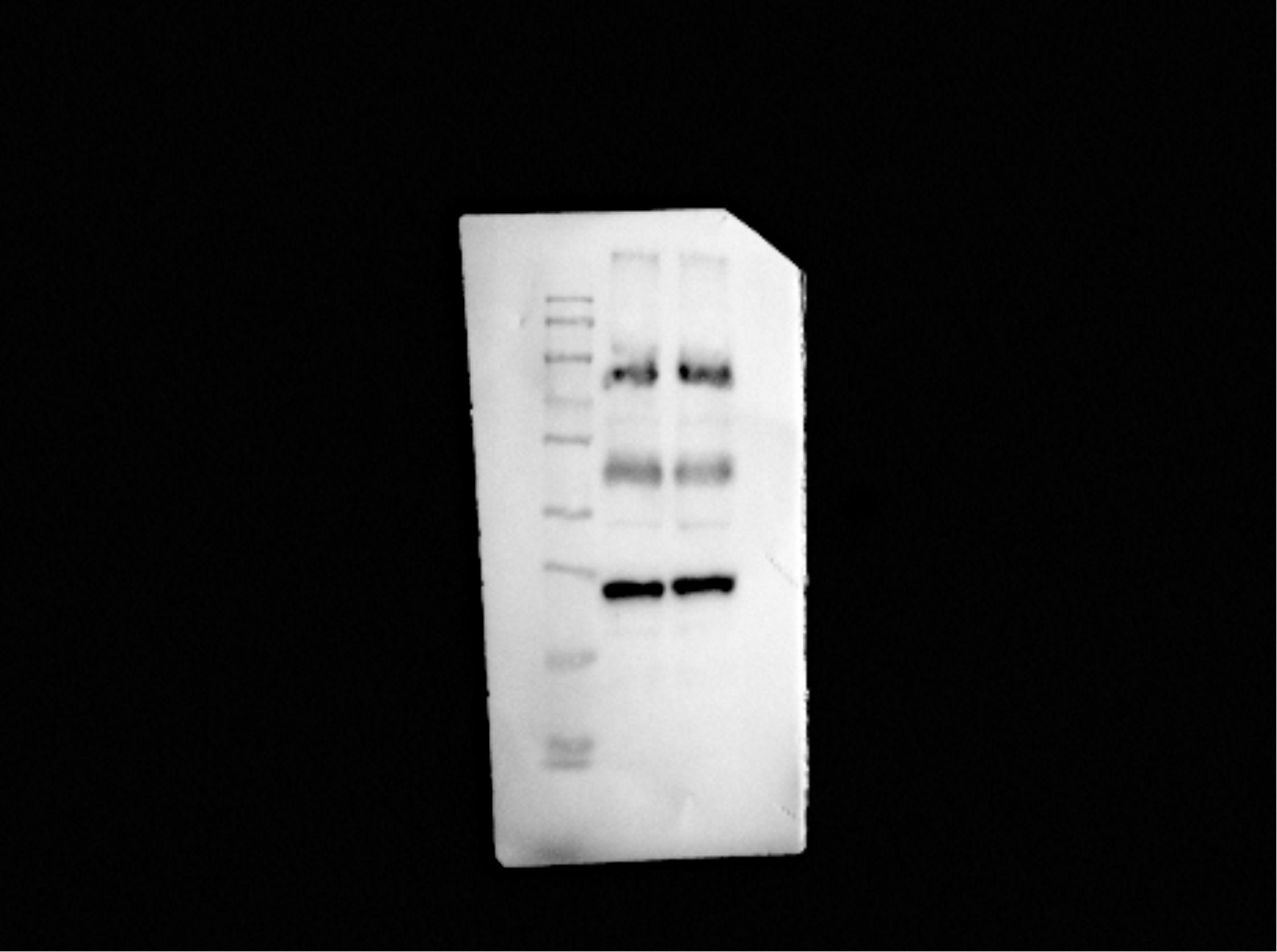

Supplement: Supplementary file 1 [file biomolecules-14-01206-s001.zip › Figure 6B-2-1.jpg]

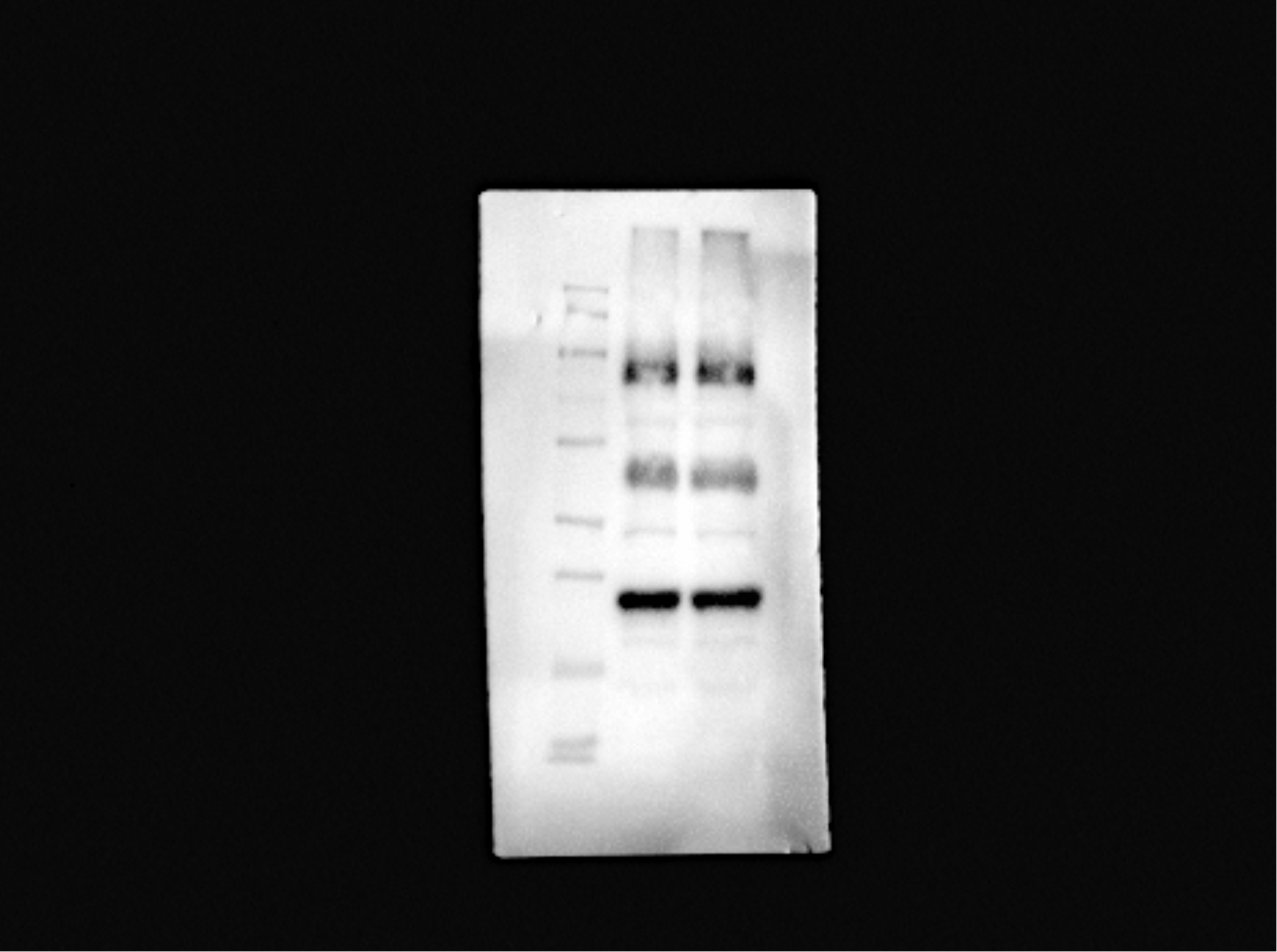

Supplement: Supplementary file 1 [file biomolecules-14-01206-s001.zip › Figure 6B-2-2.jpg]

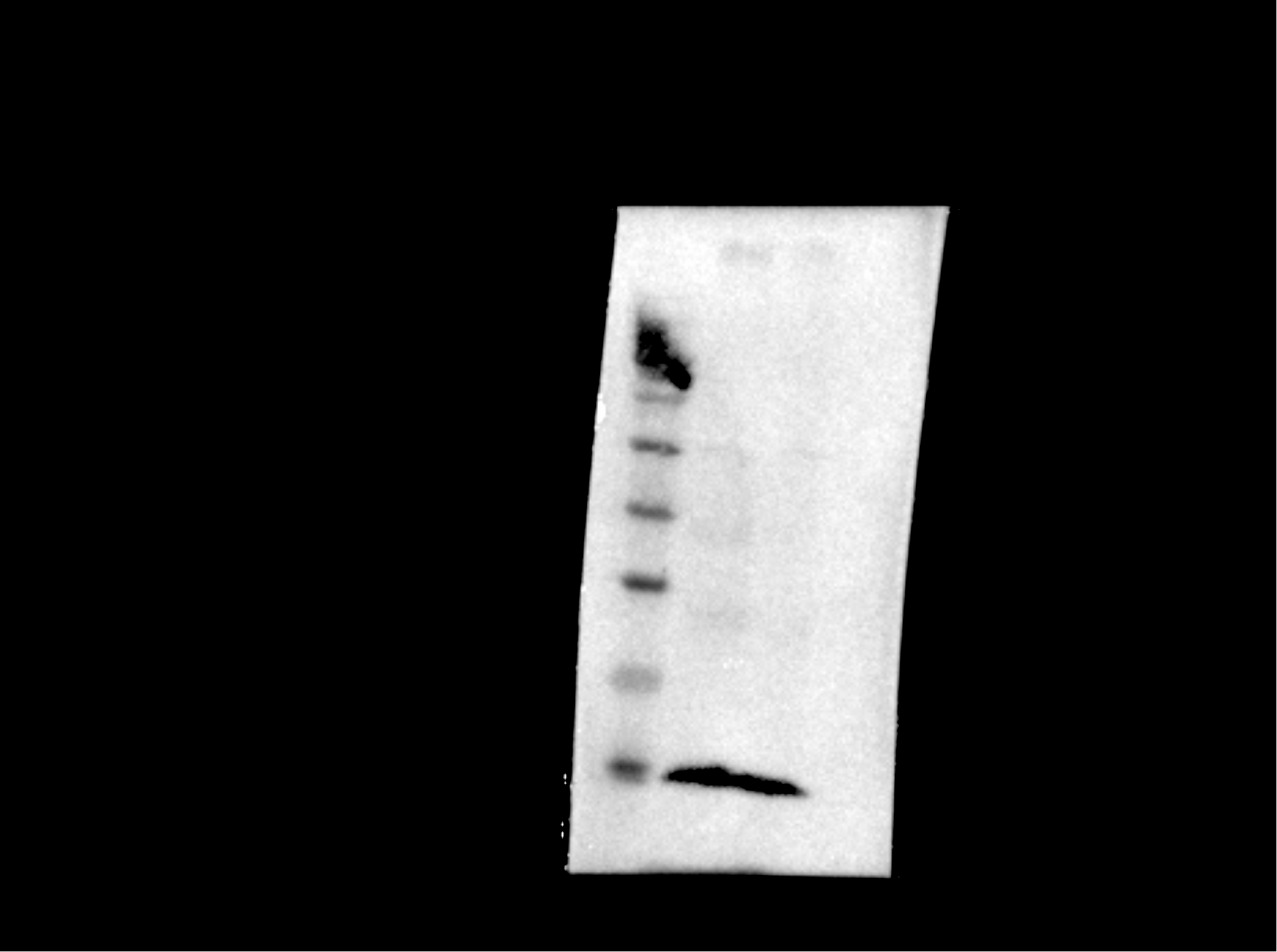

Supplement: Supplementary file 1 [file biomolecules-14-01206-s001.zip › Figure6B-1-1(new).jpg]

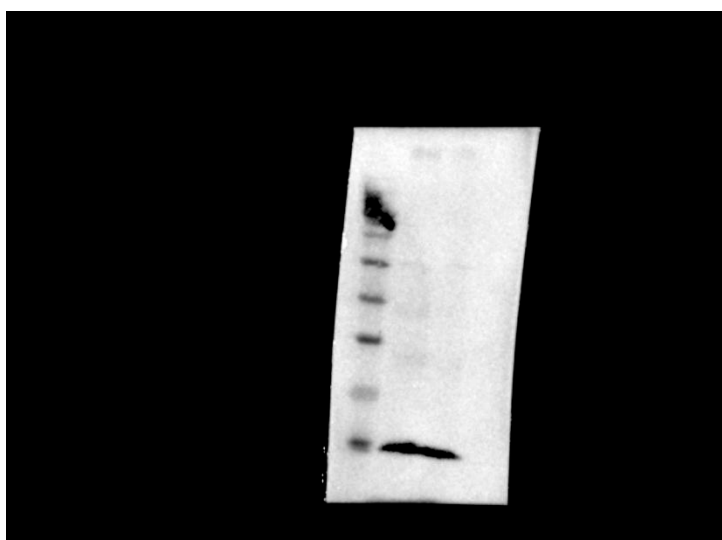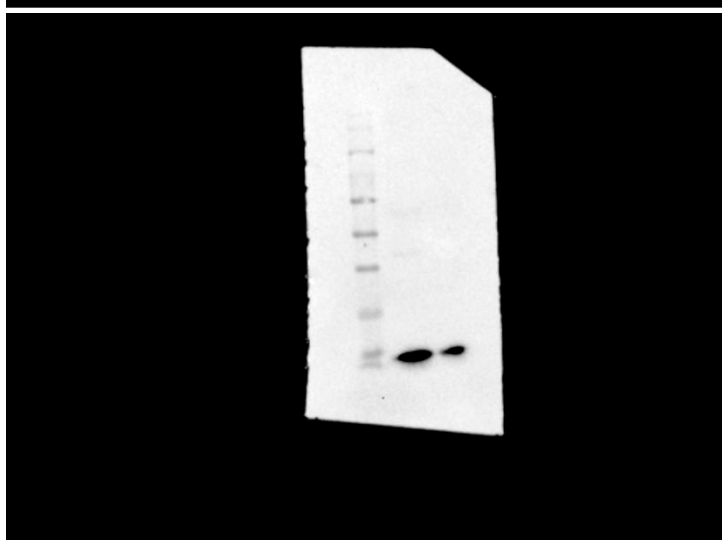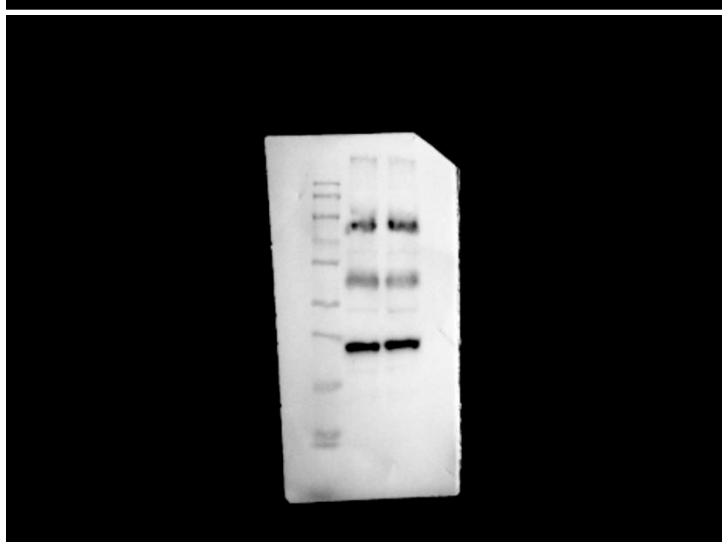

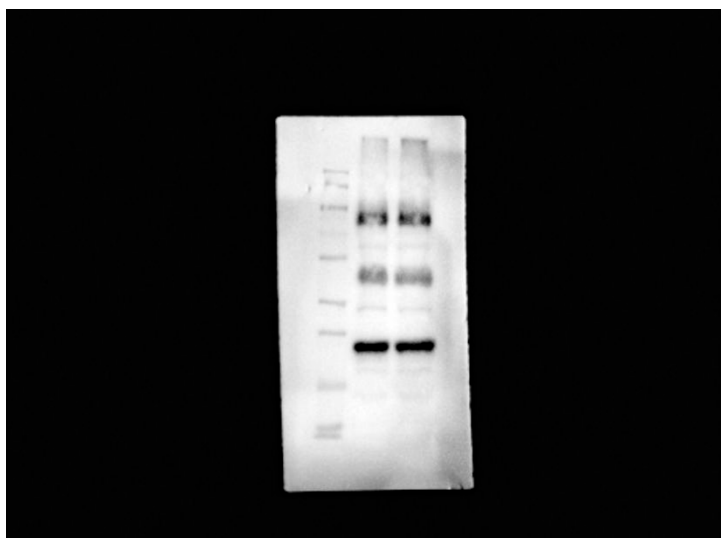

Supplement: Supplementary file 1 [file biomolecules-14-01206-s001.zip › Supplementary File 1.pdf]
